# Dyadic Synchrony and Responsiveness Within the Context of Elevated Autism Likelihood: Applying Time-Varying Effect Models

**DOI:** 10.1007/s10803-025-06891-z

**Published:** 2025-06-11

**Authors:** A. Kellerman, A. Janis, A. Piergies, S. S. Dermody, D. S. Messinger, M. Miller, A. J. Schwichtenberg

**Affiliations:** 1 Department of Human Development and Family Science, Purdue University, West Lafayette, IN USA; 2 Department of Psychiatry & Behavioral Sciences and MIND Institute, University of California, Davis, California USA; 3 Department of Psychology, Toronto Metropolitan University, Toronto, ON Canada; 4 Department of Psychology, University of Miami, Miami, FL USA

**Keywords:** Mother-infant interaction, Infant sibling, Autism, Social responsiveness, Time-varying effect model

## Abstract

The ability to engage in synchronous interactions develops in the first year, as infants learn to sequentially regulate prosocial behaviors. Difficulty developing competence in these early social building blocks is linked to later developmental concerns, including autism spectrum disorder (ASD). Currently, metrics for quantifying social competence rely primarily on mean-level indices; however, interactions are dynamic. The present study modeled change in the odds of dyadic synchrony (DS), maternal responsiveness (MR), and infant responsiveness (IR) over time to explore if temporal patterns can inform developmental monitoring. Dyads were recruited from families with at least one older child with ASD (elevated ASD likelihood, *n* = 95) or families with no history of ASD (typical ASD likelihood, *n* = 72). Theory-driven indices of dyadic synchrony and responsiveness were derived from micro-analytically coded gaze, positive affect, and vocalizations. A series of logistic time-varying effect models (TVEMs) were conducted to compare temporal changes in synchrony and responsiveness across the infant/toddler groupings of (1) elevated- vs typical-ASD likelihood and (2) typical (TYP), ASD, or other developmental concerns (Non-ASD DC). DS, IR, and MR patterns were temporally stable but lower for the elevated ASD likelihood group. Temporal patterns of DS, IR, and MR were more variable for the ASD and Non-ASD DC groups. TVEMs captured meaningful dyadic information and could be used in future studies to inform prospective monitoring and parent-mediated intervention approaches.

Early social exchanges play a pivotal role in infant development. Within the first several months of life, dyadic exchanges between parents and their infants, often referred to as an *intricate dance*, include an array of matched and mismatched repair behaviors as infants learn to sequentially regulate their reciprocal social exchanges (Feldman, 2007a; Fogel, 1993; Tronick, 1989; Tronick & Cohn, 1989). Difficulties in reciprocal social exchanges can reflect elements of postpartum depression, child maltreatment, and emerging childhood conditions (as discussed in, Feldman, 2015). Evidence of difficulties in social exchanges is especially salient in infants at elevated likelihood (EL) for autism spectrum disorder (ASD), recognizing persistent difficulties in social communication is a core diagnostic feature of ASD (American Psychiatric Association, 2013). These infants not only have a significantly higher likelihood of developing ASD themselves (Ozonoff et al., 2024), but they also receive a range of other clinically relevant outcomes, both at preschool and school ages and with some evidence of continuity between developmental periods (Charman et al., 2017; Miller et al., 2016). Extant research suggests that, while parents of children at elevated likelihood for ASD are similarly responsive compared to a typical- or low-likelihood (TL) group (Talbott et al., 2015), parents of children at elevated likelihood tend to have fewer opportunities to respond (Leezenbaum et al., 2014). Several infant siblings research groups recognize the importance of these early social building blocks with assessments of maternal sensitivity and responsiveness (Baker et al., 2010; Wan et al., 2012, 2013), infant reciprocity (Campbell et al., 2015; Harker et al., 2016), affect expressions (Cassel et al., 2007; Winder et al., 2013), infant and mother vocalizations (Leezenbaum et al., 2014; Northrup & Iverson, 2015; Paul et al., 2011; Rozga et al., 2011; Talbott et al., 2015), gaze patterns (Parladé & Iverson, 2015), and dyadic synchrony (Yirmiya et al., 2006). A few studies have noted different early social patterns in children later diagnosed with ASD. For example, Campbell et al (2015) reported lower infant social reciprocity was associated with later ADOS Comparison Scores. Similarly, Wan et al (2013) noted lower dyadic mutuality proceeded later ASD diagnoses.

Although a notable amount of research has attempted to find the meaningful ‘signal’ in early social behaviors among elevated likelihood infant siblings, no consistently replicated ‘mean-level’ markers have emerged. However, the field does agree these infants could benefit from early developmental monitoring and access to interventions. To support these ongoing developmental monitoring efforts, the present study builds upon existing representations of dyadic synchrony and responsiveness in early childhood, by examining the odds of dyadic synchrony and responsiveness at 12 months over the course of a short play session.

## Dyadic Synchrony and Responsiveness

To systematically capture the *intricate dance* between infants and their primary caregivers early in development, process-oriented microanalytic coding techniques are often used to examine who primarily leads the interaction, how responsive play partners are to one another, and the quality of their dyadic states (Feldman, 2003, 2007b). Dyadic constructs, such as synchrony and responsiveness, which are contingent on play partner’s qualifying communicative bids are well suited to distinguish social competencies as a function of possible developmental differences (e.g., dyadic coordination of vocalizations and gaze, Northrup & Iverson, 2020). Dyadic synchrony is a pattern of dyadic interactions that are mutually regulated, reciprocal, and harmonious, marked by collaboration between the child and parent. Responsiveness, while also dyadic, is distinct and refers to how a dyadic partner responds to their counterpart’s bids or social cues. While no gold standard currently exists (Leclère et al., 2014; Provenzi et al., 2018), conventional approaches to characterizing early dyadic synchrony and responsiveness in ASD research predominately include either aggregating total frequencies/durations or the proportion of time spent in a synchronous or responsive state (e.g., Siller & Sigman, 2002; Steiner et al., 2018; Yirmiya et al., 2006). However, when trying to interpret the clinical relevance of observed synchrony or responsiveness within the context of early ASD presentations, it is often unclear *how* to best target any observed group differences within an intervention setting. Aside from behavioral coding adjustments, researchers have also started utilizing advanced statistical modeling techniques to better account for the developmental nature of synchrony data (e.g., nesting; nonlinear changes), and to maximize statistical power while limiting Type 1 error rates. For example, in our previous longitudinal examinations of dyadic synchrony within elevated ASD-likelihood infants, we utilized a nonparametric repeated measures multilevel modeling framework to better represent participants with multiple data points, minimizing the total number of models built; this allowed us to interpret participants’ developmental trajectories (Kellerman et al., 2019). Though helpful to maximize opportunities for detecting robust differences, approaches such as multilevel models or latent growth curve models make assumptions relative to total linear change and average out the dependent variable across the entire timed interaction (thus ignoring temporal or changes over short periods of time). Recognizing the nature of an interaction can change over the course of just a few minutes, mean-level indices may obscure unfolding temporal processes that may be well suited to capture more distinct early ASD differences. Utilizing statistical models that are flexible enough to freely allow changes in associations across continuous time may better highlight mismatches and/or delays in social competencies to better inform ongoing developmental monitoring and intervention efforts.

To improve our current conventions and foundational understanding of reciprocal social exchanges, methodological approaches that account for unfolding dyadic nuances are needed (as discussed in Delaherche et al., 2012). Initially, social exchanges and synchrony were predominantly studied in the social sciences, but now advanced modeling approaches are common in the fields of signal processing, robotics, and machine learning (Avril et al., 2014; Leclère et al., 2016). Advanced models are being applied to try and capture the complex behavioral profiles present in reciprocal social exchanges. However, these methods often fail to capture dynamic, within-person changes in social interactions over time. Time-varying effect modeling (TVEM) offers an advantage by allowing for continuous estimation of the evolution in odds of dyadic synchrony (DS), maternal responsiveness (MR), and infant responsiveness (IR) across time. Unlike methods that focus on static or mean-level indices, TVEM accommodates nonlinearity and temporal dependencies inherent in social exchanges. For this reason, the current approach is particularly well-suited for dyadic data.

## Time-Varying Effect Modeling (TVEM)

To model the odds of social competencies changing over time, temporal associations with TVEM is well suited to highlight nuanced within-subject variability that might be missed when only capturing mean-level differences (as discussed in Lanza et al., 2016; Shiyko et al., 2014; Tan et al., 2012). The application of TVEM to the social and behavioral sciences has recently gained attention to better account for varying associations in historical time of complex data for substance use (e.g., Dermody & Shiffman, 2020; Lanza & Vasilenko, 2015; Merrill et al., 2017; Shiyko et al., 2014), as well as treatment effects (e.g., smoking cessation, Lanza & Vasilenko, 2015); Kim et al., 2021). For example, Shiyko et al. (2014) ran traditional mean-level statistical analyses (i.e., analysis of covariance, ANCOVA; multilevel modeling, MLM) and TVEM, respectively, to assess for treatment effects (as indexed by ecological momentary assessments, EMA) in lung cancer patients’ reporting on quality of life post-surgery. The repeated measures ANCOVA and MLM models revealed no significant group differences in treatment effects. Notably as a function of the aforementioned analyses, all EMA responses per patient (up to 28 unique responses) were aggregated to one average response per patient within the models. However, recognizing that patients’ recovery process and day-to-day report of distress and/or symptom burden likely varied over the two-week period (i.e., non-linear pattern), Shiyko et al. (2014) also applied TVEM to assess potential variability in time and magnitude of reported symptoms. The semi-parametric framework of the TVEM models allowed for time point comparisons (i.e., Days 1–14) of all EMA data points, which revealed significant group differences between treatment conditions by Day 4 post-surgery, providing meaningfully clinical context for supporting patients post-surgery.

Recognizing the flexibility to model associations over continuous time, TVEMs are starting to be applied to social processes as well. To the best of our knowledge, only one study to date has applied TVEM to existing dyadic observations to represent time-dependent interpersonal dynamics within married couples. Specifically, Dermody et al. (2020) assessed dimensions of interpersonal processes (i.e., affiliation; reciprocity) as they unfold over a 5-min interaction and reported cyclical patterns of change in behaviors indicative of partners “becoming more in sync” over time. To date, no ASD-specific observational studies have applied TVEM to examine temporal social processes. Employing TVEM to capture the bidirectional nature of dyadic synchrony and responsiveness during a timed interaction is well suited to provide ASD researchers with a new way to conceptualize how we may interpret early social communication differences. For example, if relatively low rates of infant responsiveness in elevated ASD-likelihood infants are observed, followed by a sharp increase in responsivity towards the end of the interaction, these findings would highlight the amount of time (or delay) it takes for elevated likelihood infants to adjust/actively respond to their play partner. With respect to ongoing parent-mediated interventions for infants at elevated likelihood for ASD (e.g., Green et al., 2017; Jones et al., 2017; Rogers et al., 2014), as prospective intervention efforts continue to target social communication skills, being able to identify subtle changes in social communication, using a model such as TVEM, may better inform how to both capture and measure progress in dyadic/social development. While TVEM is a tool for modeling time-varying odds ratios, it does not directly quantify dyadic synchrony or responsiveness. Instead, these indices were derived from behavioral coding prior to the application of TVEM, which then estimates the changing odds of coded events over time. For the purposes of this study, we have reason to believe dyadic differences may exist in the current population; however, previous studies (for summary see, Wan et al., 2019) have had limited success in finding a consistent signal to capture these differences. The use of TVEM adds temporal data to explore potential differences in another pathway.

## Current Study

The goal of the current study was to offer a new analytical consideration for assessing time-varying changes in observed play interactions in an infant sibling sample. Building on our previous work examining mean-level instances of select prosocial behaviors and dyadic constructs in elevated ASD-likelihood samples, the current study employed logistic TVEMs (a variant of TVEM for binary outcomes) to estimate the associations between dyadic synchrony and responsiveness with ASD-likelihood (as time-varying predictors) across continuous time. Specifically, we tested the following questions across two primary aims: (1) do the odds of dyadic synchrony and responsiveness change over time, and (2) do temporal changes at 12 months distinguish likelihood status or developmental outcomes. Comparing elevated- and typical-ASD likelihood groups and developmental classifications (typical, ASD, and non-ASD developmental concerns), respectively. Overall, we expected the odds of dyadic synchrony and responsivity to steadily increase over the 3 min mother–child play interaction regardless of group membership. However, we predicted the rate of change to be slower for dyads in the elevated ASD-likelihood and the developmental concerns groups (i.e., ASD; Non-ASD DC), respectively.

## Methods

### Procedure

The present study includes data from two prospective developmental monitoring studies. Both studies were conducted under the approval of their respective Institutional Review Boards, with nearly identical designs and laboratory visits every 3 to 6 months from 6 to 36 months of age. Although the two studies were similar in design, the geographic location, recruitment strategy, and assessment administration differed slightly, as described below.

Two groups of infant siblings were recruited across sites: Elevated ASD-likelihood infants from families raising at least one child with a confirmed ASD diagnosis (proband), and typical ASD-likelihood infants with no family history of ASD in 1st, 2nd, or 3rd degree relatives. The first study, Study D, was completed at the University of California, Davis and the second study, Study P, was completed at Purdue University. Probands from Study D and P were diagnosed with *DSM-5* Autism Spectrum Disorder. Study D confirmed diagnosis using the Autism Diagnostic Observation Schedule (ADOS-2; Lord et al., 2012), while Study P used copies of community diagnostic evaluations and parent-reported scores on the Social Responsiveness Scale (SRS-2; Costantino & Gruber, 2012).

As part of the final in-laboratory visit, developmental outcome groupings were assigned based on criteria developed by the Baby Siblings Research Consortium (BSRC; see Ozonoff et al., 2014). Administrations of the ADOS-2 and the Mullen Scales of Early Learning (MSEL) were used to parse out developmental group classifications. To receive an outcome classification of Typical Development (TYP), Non-ASD Development Concerns (Non-ASD DC), or ASD, children must have completed at least three visits including a final diagnostic evaluation between 24 and 36 months. The Non-ASD DC group included children with language delays, clinically relevant behavioral challenges, global developmental delay, and children exhibiting sub-threshold autism characteristics.

### Participants

One hundred sixty-seven 12-month old infants (elevated ASD-likelihood, *n* = 95 [Study D, *n* = 77; Study P, *n* = 18]; typical ASD-likelihood, *n* = 72 [Study D, *n* = 54; Study P, *n* = 18]) participated in a videotaped semi-structured play task with their mothers. One hundred fifty-four infants completed a final qualifying visit between 24 and 36 months and received one of the following outcome classifications: ASD (*n* = 14), Non-ASD DC (*n* = 33), and TYP (*n* = 107). For the purposes of this study, the 13 infants with no outcome visit classification were only included in the Aim 1 likelihood-group analyses. Select enrollment demographics are provided in Tables 1, 2 and Supplemental Table S1.

**Table 1:** Participant enrollment demographics stratified by ASD likelihood group

	Typical likelihood	Elevated likelihood	Total
N = 167			
Infant sex, *n* (%)			
Male	41 (57%)	62 (65%)	103 (62%)
Female	31 (43%)	33 (35%)	64 (38%)
Infant race, *n* (%)			
Black or African American	2 (3%)	2 (3%)	4 (2%)
Asian	0	6 (7%)	6 (4%)
White	53 (75%)	64 (69%)	117 (70%)
Multiracial	10 (14%)	15 (16%)	25 (15%)
Other^1^	5 (7%)	6 (6%)	11 (7%)
Unreported	1 (1%)	0	1 (2%)
Infant outcome, *n* (%)			
ASD	0	14 (15%)	14 (8%)
Non-ASD DC	8 (11%)	25 (27%)	33 (20%)
TYP	56 (78%)	51 (54%)	107 (64%)
No outcome	8 (11%)	5 (5%)	13 (8%)
Family characteristics			
Maternal age*^2^, *M* (SD)	31.52 (5.41)	33.71 (4.98)	*32.71* (4.98)
Maternal education*, *n* (%)			
Some high school	2 (3%)	0	2
High school or GED	5 (7%)	5 (5%)	10
Some college	9 (13%)	29 (31%)	38
College degree^3^	29 (40%)	39 (42%)	68
Some graduate school	3 (4%)	2 (2%)	5
Graduate degree^4^	23 (32%)	17 (18%)	40

^1^to include Native Hawaiian or Other Pacific Islander (*n* = 1), American Indian or Alaskan Natives (*n* = 1), or other diverse groups not specified in the provided choices (*n* = 9). ^2^Refers to biological mother’s age at child’s birth. ^3^Including Associate’s degrees and trade school degrees. ^4^Including Master’s Degree and Professional degrees (i.e., MD, PhD, JD)

^*^Maternal age and education were significantly different across the groups; wherein, mothers within the elevated likelihood group were older and had less education

**Table 2:** Participant outcome characteristics stratified by developmental classifications

	ASD (*n* = 14)	Non-ASD DC (*n* = 33)	TYP (*n* = 107)
N = 154			
Infant sex, *n* (%)			
Male	11 (79%)	18 (54%)	62 (60%)
Initial risk status, *n* (%)			
Elevated likelihood	14 (100%)	25 (76%)	51 (48%)
Typical likelihood	–	8 (24%)	56 (52%)
Outcome visit, *n* (%)			
24-month visit	–	2 (6%)	1 (1%)
30-month visit	1 (7%)	9 (27%)	6 (6%)
36-month visit	13 (93%)	22 (67%)	100 (93%)
ADOS-2 CSS^1^, *M* (SD)	8.50 (0.71)	2.29 (1.64)	1.13 (0.35)
MSEL T scores,* M* (SD)			
Fine motor skills	36.60 (7.38)	44.20 (11.26)	50.36 (9.54)
Visual reception	42.20 (12.87)	48.41 (13.75)	56.51 (11.82)
Receptive language	35.22 (13.46)	44.32 (11.85)	51.59 (8.91)
Expressive language	40.33 (14.19)	44.58 (9.84)	51.62 (8.66)

*ASD* Autism Spectrum Disorder, *Non-ASD DC* Non-ASD Development Concerns, *TYP* Typical Development. *ADOS* Autism Diagnostic Observation Schedule, *MSEL* Mullen Scales of Early Learning. ^1^Comparison (CSS) scores from ADOS-2 Total Scores as established in Esler et al., 2015; Gotham et al., 2009. Reported percentages reflect the percentage of those with outcome data

### Measures

#### Parent–Child Play Task

Mother-infant interactions were videotaped during a semi-structured free play task at the 12-month laboratory visit. Mothers were asked to play with their infants as they would at home for 3–5 min. We provided a standardized bin of age-appropriate toys—a ball, baby blanket, baby doll, set of toy phones, shape sorter, and toy car (roughly 3 inches in size with large wheels, rounded edges, and a grasping slot amendable to a palmer or raking grasp). Infants were placed in an open-top highchair and mothers were seated roughly 20 degrees off center from the child to allow for recording multiple camera angles simultaneously. Each exam room was equipped with cameras mounted behind a one-way mirror and two additional cameras in camera boxes. Cameras could be moved/adjusted without disturbing the dyad. Examiners provided materials and instructions in the room and then left to allow the dyad to play independently. The first 3 min of active engagement (not including any initial setup or seating adjustments) were microanalytically coded for infant and mother gaze (look to object, look to face), positive affect, and vocalizations following established coding rules in Ozonoff et al. (2014). Active engagement was defined as the first prosocial infant or mother behavior (e.g., mother showing a toy or infant looking to mother’s face with a smile).

For each coded behavior, trained research assistants unaware of the child’s likelihood or outcome status marked the beginning (*on*) and end (*off*) for each instance of behavior. Behaviors lasting 0.5 s or more were counted towards respective frequency and duration totals (e.g., durations > 0.5 s equal one frequency count). Total frequency counts, durations, and start and end times for each behavior were exported from the respective behavioral coding software (Study D—Noldus Observer; Study P—Mangold INTERACT). Exported data were organized sequentially in long format by participant ID, visit age, play partner, behavior, and behavior start and end times (in nanoseconds) and further processed using a Python script.

#### Dyadic Constructs

The initial development of temporally-linked dyadic constructs relied heavily on previous work (1) assessing reciprocity during play in typical development (see review, Provenzi et al., 2018), (2) distinct autism-likelihood differences in core competencies and multimodal play behaviors (see review, Wan et al., 2019), and (3) distinct autism-likelihood differences in mean-level reports of synchrony and responsiveness (e.g.,Siller & Sigman, 2002; Steiner et al., 2018). Building from the aforementioned behaviors, composites were created using the Python script to index the degree of dyadic synchrony (DS), infant responsiveness (IR), and maternal responsiveness (MR) (see Kellerman et al., 2019, 2020). Instances of DS included mutual (e.g., shared positive affect) and sequentially led prosocial behaviors (i.e., infant-led mother-follow; mother-led infant-follow). Similarly, instances of IR and MR captured the lag from one play partner’s behavior ending to the start of the other partner’s behavior (e.g., infant gaze ends, followed by the start of mother’s vocalizations). The lag from one behavior to another had to be within a three-second window to be considered responsive, in line with previous work (e.g., Kellerman et al., 2020). When a chain of behaviors occurred—for example mom smile, baby smile, baby vocalize, mom vocalize—only the two adjacent behaviors were linked. In this case, mom smile – baby smile, one count for infant responsiveness; baby vocalize – mom vocalize, one count for maternal responsiveness and two counts for dyadic synchrony. See Supplemental Table S2 for a full list of the up to 34 behavioral combinations for DS, IR, and MR.

#### Mullen Scales of Early Learning (MSEL)

The MSEL measures child abilities from birth to 68 months across five scales: gross and fine motor skills, visual reception, receptive and expressive language skills (Mullen, 1995). As part of established BSRC outcome criteria (see Ozonoff et al., 2014), standardized T-scores at children’s final laboratory visit were used to determine outcome group membership. See Table 2 for mean T Scores by outcome group.

#### Autism Diagnostic Observation Schedule, Second Edition (ADOS-2)

The ADOS-2 a standardized diagnostic tool to measure ASD symptoms across social communication and restricted and repetitive behavior domains (Lord et al., 2012). As part of each laboratory visit, a graduate student (trained by an ADOS-2 certified trainer and supervised by a research-reliable ADOS-2 investigator) or licensed clinical psychologist (certified to administer and score the ADOS-2) administered the assessment. To determine children’s developmental outcome classifications at their final laboratory visit, ADOS-2 comparison (CSS) scores (as established in Esler et al., 2015; Gotham et al., 2009) were included, following classification criteria established in Ozonoff et al. (2014). See Table 2 for mean CSS, stratified by outcome group.

#### Community Involvement

The Sleep and Developmental Studies Laboratory at Purdue University is a neurodiversity-pro space. Functionally, this means students who identify as neurodiverse are recruited and supported within the lab space. Neurodiverse students gained research experience via the data collection, coding, processing, and interpretation portions of this study. A manuscript draft was also shared with the Community-based Autism Research, Engagement, and Scholarship Youth Advisory Board (CARES YAB) at Purdue University and was adjusted based on their feedback.

### Data Analysis

All data were checked for analytic assumptions and cleaned using IBM Statistical Package for the Social Sciences (SPSS) Version 29. TVEM analyses were conducted in the Statistical Analysis System (SAS) Version 9.4 using Pennsylvania State University’s Logistic TVEM SAS Macro, which is available for download at aimlab.psu.edu/tvem (Yang et al., 2012).

#### Model Specifications

Extracted behavioral codes were re-centered at zero to establish a meaningful start time (based on the first captured infant or mother prosocial behavior) and ended 180 s following the aforementioned start time to establish a meaningful end time. Indices of DS, IR, and MR served as binary outcome variables based on the occurrence/absence (1/0) in a qualifying sequence of behaviors. Infant sex (male = 1; female = 0) and maternal education in years (Range: 9–18) were included in the main effect models as time-invariant covariates. Each TVEM model was specified with a penalized truncated power spline (commonly referred to as P Spline). Given the exploratory nature of this study, we did not have theoretical rationale for manipulating the spline and followed basic TVEM conventions (as recommended in Tan et al., 2012). See supplementary materials for sample syntax and Supplemental Figure S1 for a conceptual illustration of a TVEM for DS.

#### Aim 1

The first set of logistic TVEMs included running intercept-only models to estimate the log-odds of DS, IR, and MR, respectively, across continuous time for all dyads within each respected grouping classification (i.e., HR, LR, TYP, Non-ASD DC, and ASD). The intercept-only model for DS was specified as.

$$\ln \left[ {\frac{{p\left( {DS_{i} } \right)}}{{1 - p\left( {DS_{i} } \right)}}} \right] = \beta_{0} \left( t \right),$$

Where ln referred to the natural logarithm, p referred to the probability, ${DS}_{i}$ referred to a dummy coded variable representing an individual dyad’s $\dot{\iota }$. observed instances of dyadic synchrony (coded as 0,1 for occurrences) and ${\beta }_{0}\left(t\right)$ referred to the nonparametric coefficient function representing the log-odds of DS across continuous time (*t*). This process was repeated for both IR and MR. Results from all models were then transformed to enhance interpretation by exponentiating the coefficient functions for DS/IR/MR to represent the estimated prevalence of an event occurring. Within the context of our research questions, this allowed us to then interpret the TVEM results as the proportional rate of dyads exhibiting instances of DS/IR/MR across continuous time. Example prevalence equation provided below.

$$Prevalence \left( t \right) = \frac{{e^{{\beta_{0} \left( t \right)}} }}{{1 + e^{{\beta_{0} \left( t \right)}} }}$$

#### Aim 2

The second set of logistic TVEMs included a series of main effects models to estimate whether the log-odds of DS, IR, and MR (dichotomous outcome variables) across time significantly differ by ASD likelihood status and/or outcome classification (predictor variables). To determine whether ASD-related differences were statistically significant predictors, likelihood and outcome classifications were included as time-varying predictors. An odds ratio of 1.0 would indicate no significant association between likelihood status and DS/IR/MR (as explained in Szumila, 2010). Thus, odds ratios above 1.0 would indicate significantly *higher* odds of DS/IR/MR for individuals with higher likelihood status or developmental classification than without, whereas odds ratios below 1.0 indicate significantly *lower* odds of DS/IR/MR. Similar to the intercept-models, log-odds results from the main effect models were transformed to enhance interpretation. The transformation represents the estimated prevalence of DS/IR/MR occurring. An example equation for the initial main effects model for DS by likelihood status is provided below.

$$\ln \left[ {\frac{{p\left( {DS_{i} } \right)}}{{1 - p\left( {DS_{i} } \right)}}} \right] = \beta_{0} \left( t \right) + \beta_{1} \left( t \right)likelihood_{i} + \beta_{2} sex_{i} + \beta_{3} edu_{i} + \, \in_{i}$$

Building from the intercept-only model summarized above, ${\beta }_{1}\left(t\right){likelihood}_{i}$ was the slope function and referred to the time-varying coefficient function for likelihood group membership for each dyad. ${\beta }_{2}{sex}_{i}$ was the time-invariant coefficient function for infant sex. ${\beta }_{3}{edu}_{i}$, referred to the time-invariant coefficient function for maternal education (in years) for each dyad. And the error term, ${\epsilon }_{i}$, represented dyad’s deviation from their expected instances of DS (i.e., difference between observed and expected DS). Additional main effects models were specified for IR and MR, as well as by developmental classifications for DS, IR, and MR.

## Results

Likelihood status models for DS included a total of 16,383 observations of DS (elevated ASD likelihood = 11,886; typical ASD likelihood = 9341). Developmental classification models included 19,646 observations (ASD = 1762; Non-ASD DC = 3,960; TYP = 13,924). Total summary descriptive statistics of DS, IR, and MR by likelihood and developmental classification are provided in Table 3. As per conventions with TVEM, the model results are predominately presented in figures because the coefficients are estimated for continuous time and therefore yield coefficients for each moment in time that cannot be adequately summarized in a concise table. However, abridged summaries of corresponding exponentiated coefficients at 20-s intervals from 0 – 180 s are provided in Table 4 (for DS) and Supplementary Tables S3, S4, and S5 for overall, IR and MR.

**Table 3:** Dyadic synchrony and responsiveness composite frequencies stratified by likelihood and developmental classification

	EL	TL	ASD	Non-ASD DC	TYP
Dyadic synchrony					
Mean, SD	42.79 (19.7)	51.25 (19.5)	44.69 (16.3)	37.34 (16.2)	49.61 (21.0)
Range, Min–Max	6–90	12–104	16–89	6–80	6–104
Infant responsiveness					
Mean, SD	22.60 (10.1)	26.89 (9.8)	23.54 (9.2)	20.06 (8.4)	25.93 (10.6)
Range, Min–Max	4–48	8–53	8–48	4–41	5–53
Maternal responsiveness					
Mean, SD	22.65 (9.8)	27.14 (9.5)	23.92 (7.9)	19.56 (8.2)	26.28 (10.3)
Range, Min–Max	4–47	6–52	12–46	4–41	6–52

*EL* Elevated Likelihood, *TL* Typical Likelihood, *ASD* Autism Spectrum Disorder, *Non-ASD DC* Non-ASD Development Concerns, *TYP* Typical Development

**Table 4:** Prevalence of dyads exhibiting dyadic synchrony during a play interaction

	ASD Likelihood Status		Developmental Classification		
Prevalence (CI)	EL	TL	ASD	Non-ASD DC	TYP
Time (sec)					
0	.403 (.363–.444)	.418 (.380–.458)	.350 (.251–.464)	.373 (.320–.430)	.488 (.443–.532)
20	.351 (.324–.379)*	.393 (.366–.420)	.352 (.281–.429)	.309 (.270–.350)*	.352 (.320–.386)
40	.325 (.299–.353)*	.382 (.353–.411)	.346 (.276–.423)	.283 (.241–.329)*	.381 (.351–.411)
60	.318 (.293–.345)*	.381 (.353–.410)	.332 (.271–.400)	.282 (.242–.325)*	.379 (.350–.409)
80	.323 (.300–.347)*	.387 (.360–.414)	.315 (.255–.382)	.294 (.258–.333)*	.369 (.341–.398)
100	.334 (.310–.358)*	.394 (.366–.422)	.303 (.242–.372)	.311 (.274–.352)*	.379 (.353–.407)
120	.344 (.319–.370)*	.398 (.369–.429)	.304 (.245–.371)	.323 (.280–.370)*	.383 (.354–.414)
140	.348 (.322–.374)*	.396 (.368–.425)	.320 (.266–.379)*	.318 (.273–.366)*	.386 (.359–.413)
160	.338 (.311–.367)*	.382 (.356–.410)	.343 (.299–.390)	.287 (.239–.340)*	.395 (.364–.427)
180	.311 (.267–.359)*	.354 (.307–.404)	.356 (.279–.440)	.226 (.156–.315)*	.311 (.261–.365)

*CI* confidence interval, *EL* elevated ASD likelihood, TL typical ASD likelihood, *ASD* autism spectrum disorder, *Non-ASD DC* non-autism developmental concerns, *TYP* typical development. The provided exponentiated coefficients and CIs assess the odds of synchrony at a given point in the interaction. This table is provided for descriptive purposes only – overall TVEM interpretation is based on the entire 180 s sample (see Figures). *Significant group differences at this sample point in time based on 95% CIs–EL compared to TL, ASD compared to TYP, Non-ASD DC compared to TYP

### Aim 1—Change Over Time

**Fig. 1:**
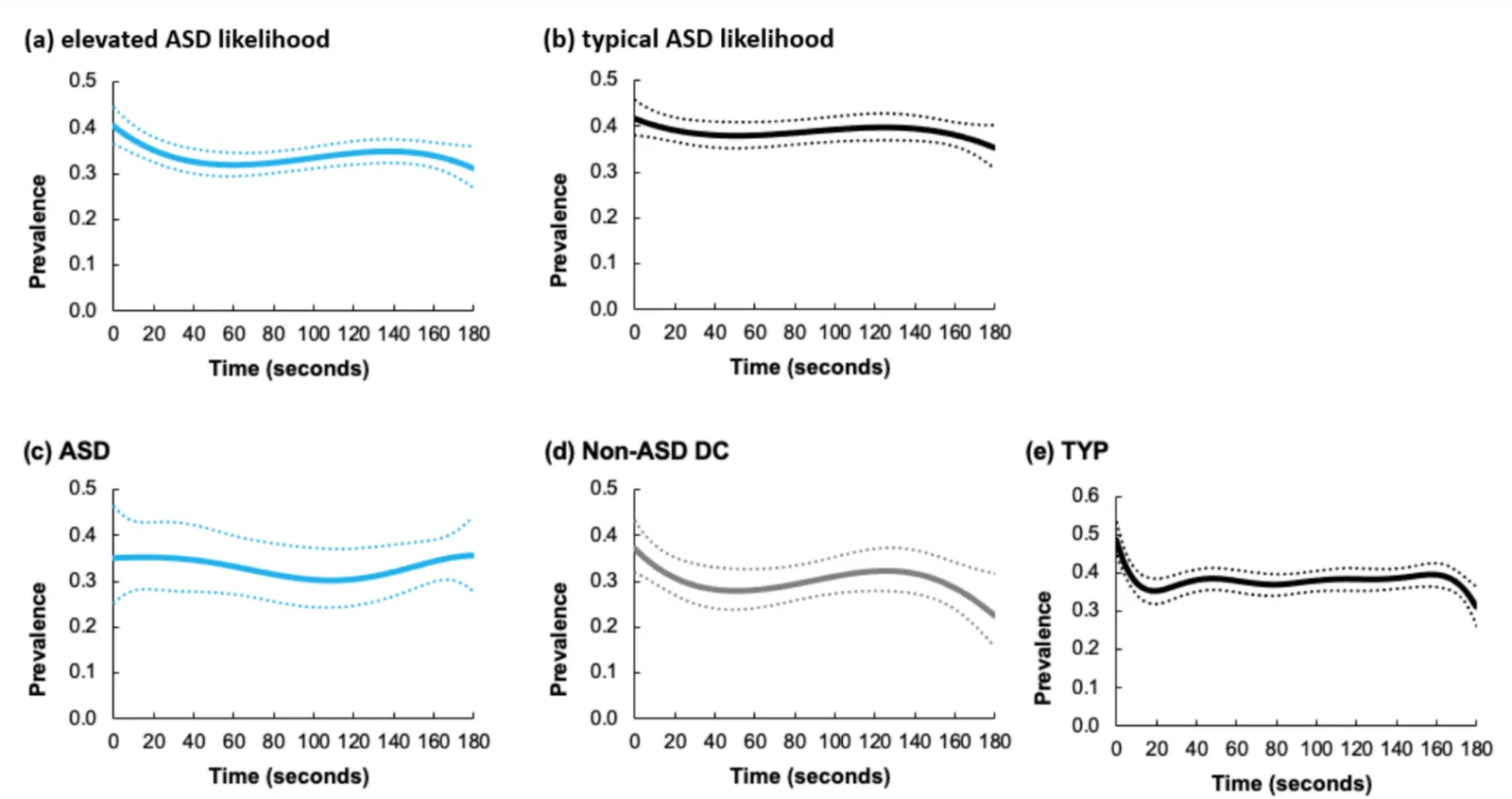
Estimated prevalence of DS (solid lines) across time. Dotted lines represent 95% confidence intervals. The X-axis is labeled with time in seconds that instances of DS occurred for a respected group during the 3 min coded play interaction. Aim 1: Proportion of dyads engaging in dyadic synchrony during a play interaction, estimated by intercept-only TVEM

To descriptively examine whether rates of DS, IR, and MR steadily increase over time, a series of intercept-only TVEM models were conducted, within each respected group classification. For DS, dyads in the typical ASD likelihood and TYP groups, respectively, exhibited the highest overall odds of DS (typical ASD likelihood: 38.9%; TYP: 37.9%) followed by comparable odds in the elevated ASD likelihood (33.7%) and ASD (33.0%) groups, and lastly 30.1% in the Non-ASD DC group (Fig. 1). Specific to likelihood status, dyads in the elevated ASD likelihood group exhibited slight variability across time between their highest odds occurring at the start of the interaction (40.3%, CI: 0.36, 0.44) and their lowest at the end (31.1%, CI: 0.27, 0.36), as illustrated in Fig. 1a. For dyads in the typical ASD likelihood group, DS was stable for the majority of the interaction (ranging between 38–39%) before decreasing to 35.4% (CI: 0.31, 0.40) within the last 10 s (Fig. 1b). With respect to developmental classifications, dyads in the ASD group exhibited the most variability across time, as evident by corresponding wide confidence intervals across the entire interaction (Fig. 1c). In addition, distinct from the other groups, dyads in the ASD group exhibited the highest DS at the start (35.0%, CI: 0.25, 0.46) and end (35.6%, CI: 0.28, 0.44) of the play interaction, with the lowest (30.8%) occurring during the middle of the interaction, from approximately 90 to 130 s. Dyads in the Non-ASD DC group also exhibited more fluctuation in DS across time, with rises and falls occurring approximately every 60 s, following the first fall at 50 s (28.0%, CI: 0.24, 0.33), before the final fall beginning at 160 s (28.7%, CI: 0.24, 0.34). Lastly, dyads in the TYP group predominately followed a stable, yet slowly decreasing pattern, with the exception of a drop in response rate within the first 20 s from 48.8% (CI: 0.44, 0.53) to 35.2% (CI: 0.32, 0.39). A summary of DS results by group membership are illustrated in Fig. 1 and an abridged summary of exponentiated coefficient functions are presented in Table 4.

**Fig. 2:**
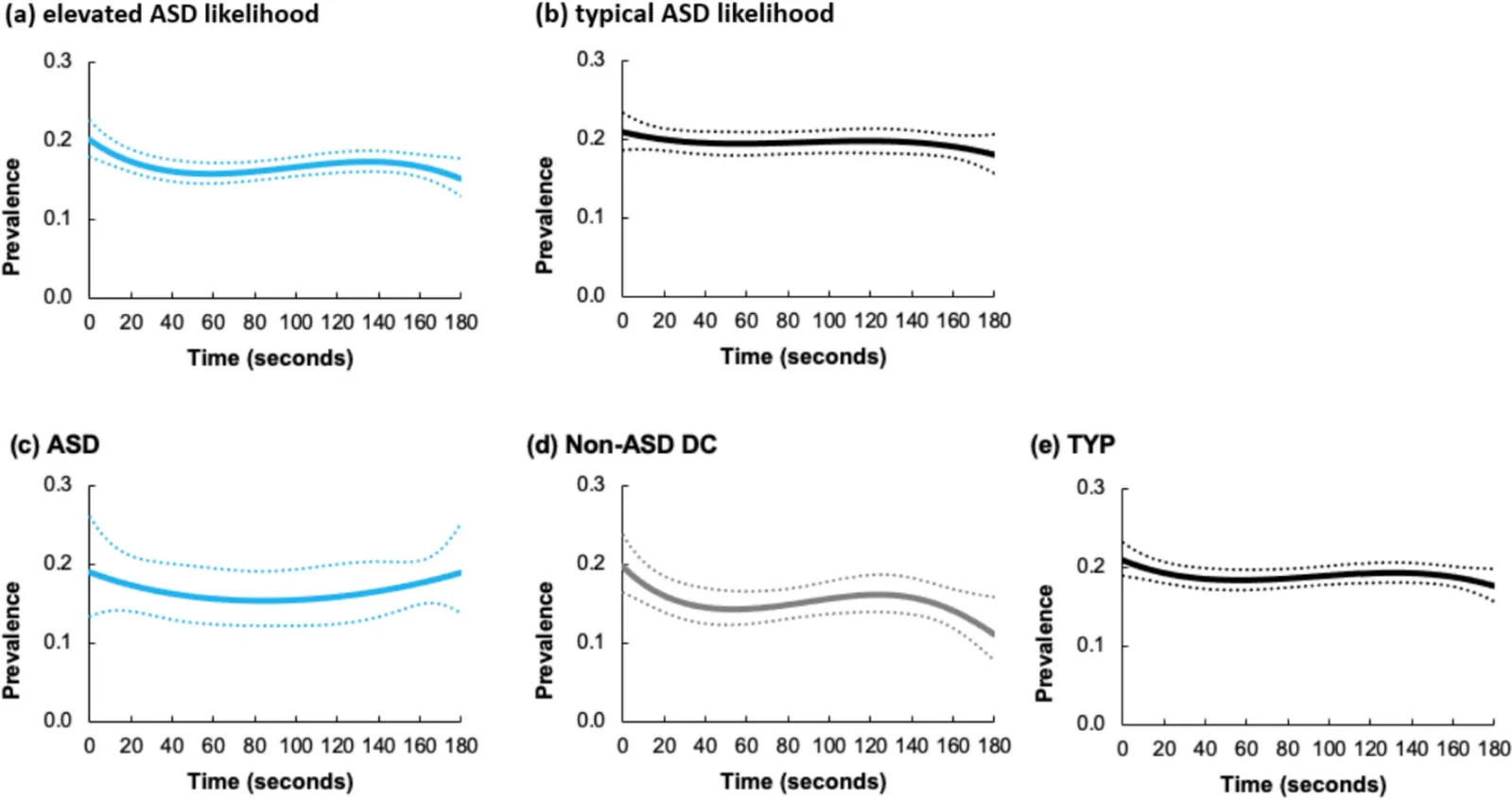
Estimated prevalence of IR (solid line) across time. Dotted lines represent 95% confidence intervals. The X-axis is labeled with time in seconds that instances of IR occurred for each respected group during the 3 min coded play interaction. Aim 1: Proportion of dyads engaging in infant responsiveness during a play interaction, estimated by intercept-only TVEM

**Fig. 3:**
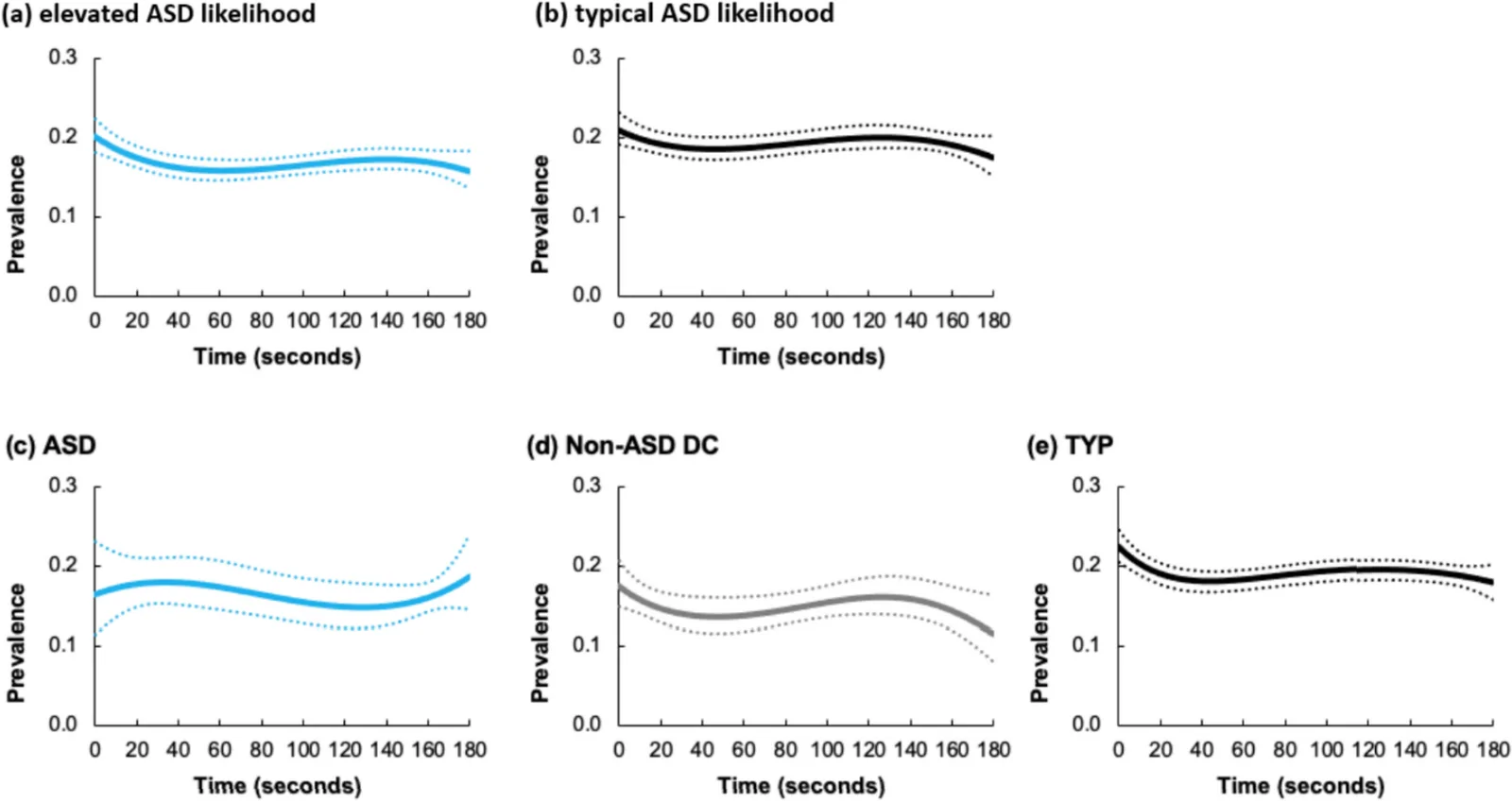
Estimated prevalence of MR (solid line) across time. Dotted lines represent 95% confidence intervals. The X-axis is labeled with time in seconds that instances of MR occurred for a respected group during the 3 min coded play interaction. Aim 1: Proportion of dyads engaging in maternal responsiveness during a play interaction, estimated by intercept-only TVEM

With respect to the prevalence of responsiveness per each respected group, IR and MR followed comparable patterns with an average prevalence rate of ~ 20% throughout the interaction (Figs. 2 and 3). For IR, the most variability was observed for the ASD and Non-ASD DC groups, respectively. For dyads in the ASD group, IR was the highest at the start of the interaction (19.0%, CI: 0.13, 0.26), followed by a subtle decline over the next 100 s to 15.4% (CI: 0.12, 0.19), and ended at a comparable rate to the beginning of the interaction (18.9%, CI: 0.14, 0.25). However, dyads in the Non-ASD DC group demonstrated a subtle rise and fall pattern (similar to DS) such that dyads’ IR declined from their initial start at 19.9% (CI: 0.16, 0.24), followed by a subtle uptick close to the midpoint of the interaction before subtly declining to their lowest rate at the end of the interaction (11.2%, CI: 0.08, 0.16). Similarly with MR, the ASD and Non-ASD DC dyads demonstrated the most variability in response rates and followed the same general pattern as their respected IR rates (Fig. 2). Abridged summaries of exponentiated coefficient functions for IR and MR, respectively, are presented in Supplementary Tables S4 and S5.

### Aim 2—Group Differences

To assess whether significant likelihood status and/or developmental classification group differences existed in DS, IR, and MR, a series of main effects TVEMs were conducted with likelihood and developmental classification, respectively, included as time-varying predictors and time-invariant terms for infant sex and maternal education.

**Fig. 4:**
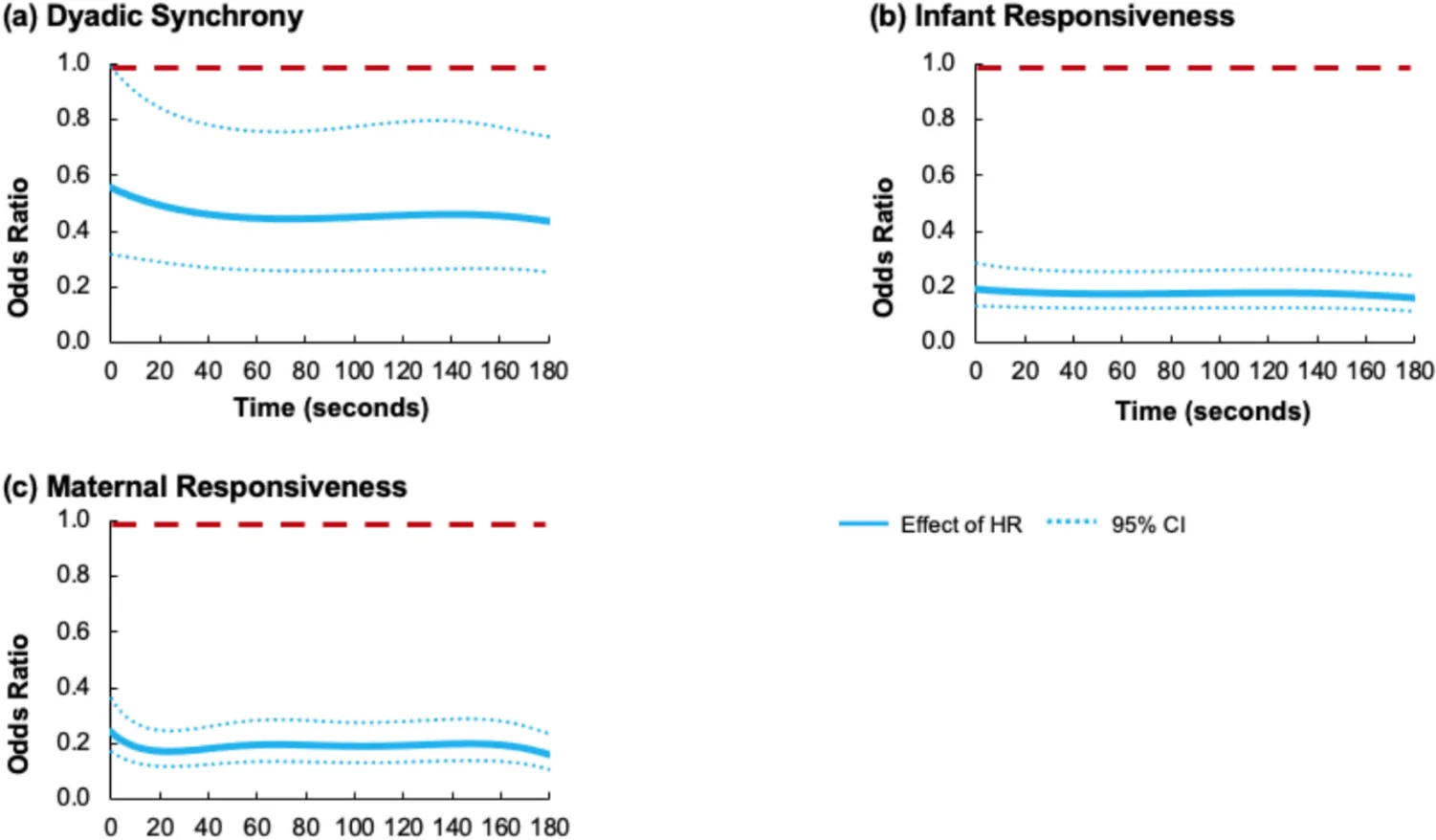
Odds ratio corresponding to the change in the odds of DS/IR/MR being associated with elevated ASD likelihood dyads across a 3 min play interaction. The solid blue line represents the odds ratio associated with being elevated ASD likelihood across time. The dashed blue lines represent the 95% confidence interval (CI). An odds ratio of 1.0 (dashed red line) indicates no significant association between risk status and DS/IR/MR. Odds ratios above 1.0 indicate significantly higher odds of DS/IR/MR. Odds ratios below 1.0 indicate significantly lower odds of DS/IR/MR. Aim 2: Time-varying odds ratios showing the effect of being elevated likelihood on indices of social engagement

Specific to likelihood status, dyads in the elevated ASD likelihood group were at significantly lower odds of exhibiting instances of DS, IR, and MR relative to dyads in the typical ASD likelihood group throughout the 180-s coded interaction (Fig. 4). However, as depicted in Fig. 4, minimal fluctuations in the log-odds of DS, IR, or MR at respected times across the interaction were observed, and for DS, large CI bands were evident throughout, ranging from 0.99 to 0.26. Specific to DS, the largest decline occurred within the first minute ranging from the first 2 s (OR = 0.55, CI = 0.32–0.97) to 67 s (OR = 0.44, CI = 0.26–0.76). For IR, the log-odds for the effect of elevated ASD likelihood were stable at an odds ratio of ~ 0.2 throughout the interaction. Specific to MR, the largest trough occurred at approximately 24 s (OR = 0.17, CI = 0.12–0.25). Infant sex was not a significant time-invariant covariate. However, maternal education was significant for the DS and IR likelihood status models, such that higher education (in years) was associated with higher rates of DS (*p* = 0.02) and IR (*p* = 0.02).

**Fig. 5:**
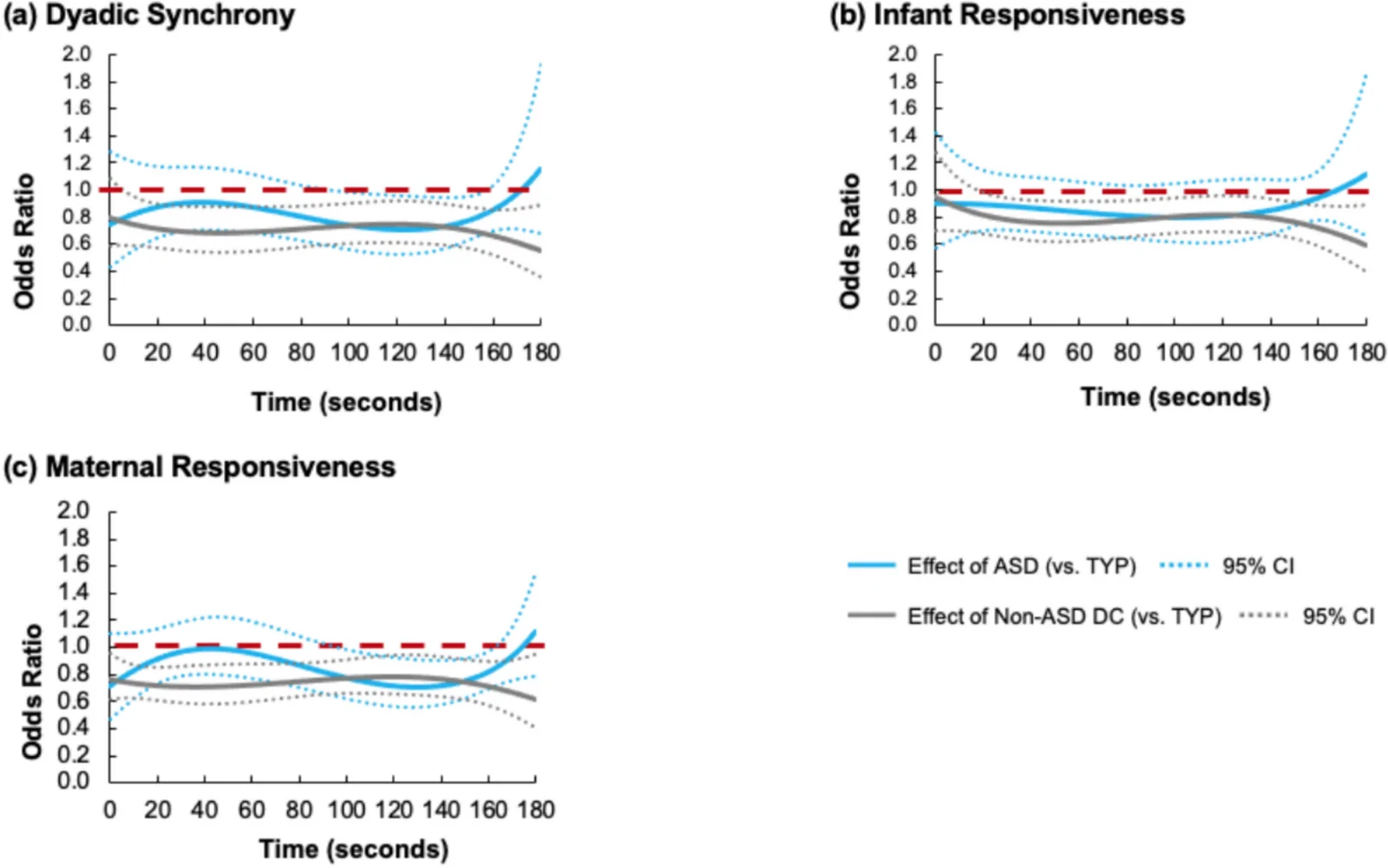
Odds ratio corresponding to the change in the odds of DS/IR/MR being associated with developmental concern classifications across a 3 min play interaction. The solid blue line represents the odds ratio associated with the effect of ASD (vs. TYP) across time. The solid gray line represents the odds ratio associated with the effect of Non-ASD DC (vs. TYP) across time. The dashed lines represent the 95% confidence intervals (CI). An odds ratio of 1 (represented as the dashed red line) indicates no significant association between classification and DS/IR/MR. Odds ratios above 1.0 indicate significantly higher odds of DS/IR/MR. Odds ratios below 1.0 indicate significantly lower odds of DS/IR/MR. Aim 2: Time-varying odds ratios showing the effect of developmental concern classifications on indices of social engagement

With respect to developmental classifications, group differences between ASD, Non-ASD DC, and TYP dyads were assessed by specifying dummy-coded indicators with TYP as the reference group. Overall, ASD dyads (compared to TYP), only exhibited significantly lower odds of DS from 103.6 to 152.7 s, with the largest trough occurring at approximately 124 s (OR = 0.71, CI = 0.53–0.96). Whereas Non-ASD DC dyads (compared to TYP) exhibited significantly lower odds of DS for the vast majority of the interaction (10.9–180.0 s) with the largest trough occurring at approximately 45 s (OR = 0.69, CI = 0.54–0.87; Fig. 5a). In terms of responsiveness, no significant differences in log-odds of IR were observed for ASD compared to TYP dyads. In the non-ASD DC group (when compared to TYP), significantly lower odds of IR occurred at two time point ranges (27.3–107.3 s; 138.2–180.0 s), with the largest drop at approximately 176 s (OR = 0.62, CI = 0.44–0.88; Fig. 5b). For MR, ASD dyads exhibited significantly lower odds from 110.9 to 156.4 s, whereas Non-ASD DC dyads’ rates of MR were significantly lower for the majority of the interaction (1.8–178.2 s; Fig. 5c). For ASD (compared to TYP), the largest dip in MR occurred towards the end of the interaction at ~ 127 s (OR = 0.71, CI = 0.56–0.91), whereas for Non-ASD DC the largest drop occurred towards the beginning at 30 s (OR = 0.71, CI = 0.51–0.87). It should be noted that while these results are statistically significant, the *clinical* significance is unknown. In terms of time-invariant covariates, only maternal education was significant on select models. Specifically, higher educational attainment (in years) was associated with significantly more instances of IR (*p* = 0.001) and MR (*p* = 0.02).

## Discussion

The present study aimed to address social/dyadic complexity by leveraging TVEMs to document how dyadic synchrony and responsiveness ratios change over time, within the context of elevated ASD likelihood. This study addressed (Aim 1) stability over time (across the 180 s observation) and (Aim 2) group differences in the likelihood of DS, MR, or IR during play. In sum, dyads were fairly stable in their DS, MR, and IR over time regardless of group status. Large changes were not apparent in our short observations. For group differences, the findings are more nuanced but reflected more variability in the EL, ASD, and Non-ASD DC groups. Similarly, less DS, MR, and IR were apparent in the EL (when compared to TL) and the Non-ASD DC (when compared to TYP) groups. Surprisingly, the ASD group only demonstrated lower rates of DS, MR, and IR at select intervals (when compared to the TYP group). In the following sections we’ll ground these findings within the existing literature, organized by study aim.

### Aim 1—Change Over Time

Building on previous research (e.g., Dermody et al., 2020), we hypothesized notable shifts in DS, MR, and IR over the course of our 3-min play observation. Given the novel application of TVEM, we were unsure if dyads would build in their synchrony over time (our initial hypothesis), show an oscillatory pattern, or decrease over time. Given that this study was the first to apply TVEMs in an infant sibling design there is limited direct literature to ground this work in but the larger literature on dyadic behaviors may provide some context. The “serve and return” phenomenon is a cornerstone of early social development and research consistently documents how synchrony and contingent responsivity are associated with early-life skills such as language and self-regulation (Eulau & Hirsh-Pasek, 2024; Masek et al., 2021; Scholtes et al., 2021). Contingent responsive social exchanges often follow a sing-song pattern with periods of engagement followed by periods of disengagement/regulation (for a review see Feldman, 2007b). Given this, the finding of stability over time in the present study was unexpected. The overall stable pattern may be a reflection of the coding system, wherein engagement behaviors (e.g., looking to the mother’s face) and disengagement behaviors (e.g., saying “no”) would both be coded without regard to the qualitative nature of the bid/exchange. Future studies could probe at this qualitative explanation by incorporating coding systems that capture not only the frequency of synchronous or responsive behaviors but also elements about their purpose (to engage, respond, disengaged, etc.).

### Aim 2—Group Differences

Differences in elements of synchrony and responsiveness within elevated likelihood samples have been documented in previous studies (Campbell et al., 2015; Wan et al., 2012, 2013; Yirmiya et al., 2006). With some noting lower synchrony or more parent directiveness in their EL groups. Within this sample, we did see less overall synchrony and responsiveness with the EL group, but we are hesitant to attribute this to maternal behaviors as our data clearly demonstrates the dyadic nature of our coding system wherein lower DS, IR, and MR were apparent.

Overall our likelihood group results echo previous prospective infant-sibling findings wherein elevated likelihood group differences are often primarily driven by social difficulties within the Non-ASD DC group. This is especially evident in our previous studies encompassing a subset of the current sample at 12 months, in which the Non-ASD DC group primarily drove mean-level differences in IR, and distinct repertories of responsive behaviors (Kellerman et al., 2020). Although recognizing the Non-ASD DC group primarily included children with language-specific concerns, this finding may further highlight the active role language development plays in our indices of dyadic synchrony and responsiveness. In fact, infants (regardless of group membership) with higher DS, IR, and/or MR at 12 months scored significantly higher on the MSEL receptive and expressive language subscales at 36 months. However, recognizing our limited sample sizes of ASD (*n* = 14) and Non-ASD DC (*n* = 33) infants, this connection should not be overinterpreted.

Our findings provide an initial glimpse into how analyzing social processes over continuous time may provide more context beyond our current conventions of solely aggregating behavior totals. First, while the Non-ASD DC group (compared to the TYP group) consistently exhibited significantly lower log-odds of DS, IR, and MR, across the vast majority of the interaction, they also exhibited the most fluctuation in their unfolding rates of synchrony and responsivity. Specifically, their lowest DS and MR occurred within the first minute, whereas their lowest IR occurred within the last 20 s (of the 3-min interaction). Recognizing our MR composite was derived from infant-initiated prosocial behaviors, and our IR composite is comprised of mother-initiated behaviors, the aforementioned responsiveness dips may be indicative of needed adjustment periods for infants. For the Non-ASD DC group, it’s conceivable that infants may have needed the first 30 s to ‘warm-up’ to the task/environment before consistently initiating prosocial exchanges (as evidenced by the low MR) and similarly, may have socially/cognitively exhausted themselves towards the end of the interaction (as evidenced by the drop in IR). Alternatively, for the ASD group (compared to the TYP group) no significant differences in IR were observed; however, for DS and MR, the lowest rates did not occur until approximately two minutes into the interaction. Meaning unlike the patterns observed for the Non-ASD DC group, the ASD group demonstrated fairly comparable rates to their typically developing peers. With respect to MR, it’s similarly possible that infants in the ASD group experienced social burnout/exhaustion after almost 2 min of play with their mothers, contributing to a drop in their initiations (as evidenced by the drop in MR). Unfortunately, given the small number of children with ASD within this sample and the relatively low levels of individual behaviors (see Table 5) we felt it was not advisable to test this directly within the present sample.

**Table 5:** Descriptives of qualifying region-level dyadic sequences with maximum response timing for Infant and Maternal Responsiveness

		Elevated ASD likelihood	Typical ASD likelihood	ASD	Non-ASD DC	TYP
Infant responsiveness, M (SD)						
Mother-led look face…	Followed by infant look face	2.09 (2.04)	2.97 (3.10)	2.29 (2.20)	1.38 (1.68)	2.74 (2.71)
	Followed by infant look object	1.16 (1.38)	1.39 (1.25)	0.86 (1.17)	0.84 (0.99)	1.44 (1.44)
	Followed by infant positive affect	0.74 (1.09)	0.56 (0.95)	1.00 (1.18)	0.37 (0.71)	0.64 (1.03)
	Followed by infant vocalizations	1.31 (1.57)	1.38 (1.57)	1.71 (1.59)	0.78 (0.91)	1.52 (1.70)
Mother-led look object…	Followed by infant look face	0.63 (1.08)	1.06 (1.16)	0.86 (1.75)	0.28 (0.58)	0.99 (1.15)
	Followed by infant look object	1.52 (1.49)	2.03 (1.81)	1.29 (1.38)	1.03 (1.12)	2.03 (1.79)
	Followed by infant positive affect	0.19 (0.57)	0.15 (0.40)	0.36 (0.84)	0.22 (0.42)	0.14 (0.47)
	followed by infant vocalizations	0.97 (1.31)	1.07 (1.29)	0.64 (0.75)	0.47 (0.98)	1.24 (1.36)
Mother-led positive affect…	Followed by infant look face	0.61 (0.91)	0.65 (0.76)	0.57 (0.94)	0.50 (0.80)	0.69 (0.87)
	Followed by infant look object	0.94 (1.12)	1.14 (1.33)	0.64 (0.63)	1.19 (1.51)	1.02 (1.18)
	Followed by infant positive affect	0.45 (0.65)	0.66 (0.83)	0.57 (0.76)	0.25 (0.44)	0.60 (0.78)
	Followed by infant vocalizations	0.62 (1.02)	0.42 (0.84)	0.50 (0.94)	0.22 (0.55)	0.64 (1.00)
Mother-led vocalizations…	Followed by infant look face	2.83 (2.49)	3.44 (2.79)	2.29 (2.30)	1.88 (2.12)	3.57 (2.66)
	Followed by infant look object	2.28 (2.19)	3.06 (2.35)	2.43 (2.68)	1.34 (1.70)	3.04 (2.30)
	Followed by infant positive affect	0.81 (0.97)	0.66 (0.99)	0.93 (1.00)	0.66 (1.07)	0.75 (0.94)
	Followed by infant vocalizations	1.69 (1.99)	1.58 (1.64)	1.71 (1.73)	1.13 (1.21)	1.83 (1.97)
Maternal Responsiveness, M (SD)						
Infant-led look face…	Followed by mother look face	0.95 (1.34)	1.51 (1.76)	1.36 (2.17)	0.63 (1.04)	1.36 (1.61)
	Followed by mother look object	0.76 (1.15)	0.85 (1.31)	0.43 (0.65)	0.38 (0.83)	0.94 (1.31)
	Followed by mother positive affect	1.09 (1.12)	1.44 (1.44)	1.21 (1.25)	0.84 (1.11)	1.42 (1.39)
	Followed by mother vocalizations	1.91 (2.05)	2.55 (2.19)	1.43 (1.60)	1.22 (1.95)	2.53 (2.16)
Infant-led look object…	Followed by mother look face	1.61 (1.40)	2.28 (2.01)	1.43 (1.51)	1.13 (0.98)	2.16 (1.79)
	Followed by mother look object	2.80 (2.31)	3.51 (2.72)	2.86 (2.45)	1.56 (1.66)	3.60 (2.57)
	Followed by mother positive affect	0.57 (0.87)	0.70 (0.95)	0.57 (0.94)	0.31 (0.59)	0.71 (0.97)
	Followed by mother vocalizations	2.58 (2.04)	3.25 (2.66)	2.43 (1.51)	2.28 (2.43)	3.14 (2.34)
Infant-led positive affect…	Followed by mother look face	0.19 (0.47)	0.32 (0.53)	0.29 (0.61)	0.25 (0.51)	0.25 (0.50)
	Followed by mother look object	0.34 (0.59)	0.44 (0.77)	0.64 (0.75)	0.13 (0.34)	0.40 (0.73)
	Followed by mother positive affect	0.54 (0.93)	0.45 (0.84)	0.71 (1.33)	0.50 (0.95)	0.48 (0.84)
	Followed by mother vocalizations	0.79 (1.08)	0.69 (1.10)	1.43 (1.79)	0.50 (0.88)	0.73 (1.01)
Infant-led vocalizations…	Followed by mother look face	1.03 (1.37)	1.04 (1.33)	1.00 (1.18)	0.47 (0.95)	1.19 (1.32)
	Followed by mother look object	1.18 (1.52)	1.27 (1.47)	0.79 (0.98)	0.72 (0.89)	1.45 (1.65)
	Followed by mother positive affect	0.49 (1.01)	0.38 (0.68)	0.36 (0.50)	0.25 (0.67)	0.49 (0.95)
	Followed by mother vocalizations	2.15 (2.48)	2.00 (2.20)	2.29 (2.70)	0.97 (1.12)	2.46 (2.52)

*ASD* Autism Spectrum Disorder, *Non-ASD DC* Non-ASD Development Concerns, *TYP* Typical Development

Though our aforementioned interpretation of intent behind the observed dips in IR and MR is speculative in nature, being able to explore whether the amount of time (or delay) it takes for infants at elevated likelihood for ASD to adjust/actively respond to their play partner could be highly relevant to ongoing pre-diagnosis/pre-emptive parent-mediated interventions targeting social indices to optimize children’s development trajectories (e.g., Davis et al., 2022; Green et al., 2017; Tanner & Dounavi, 2020; Whitehouse et al., 2019).

In reflecting on our ASD and Non-ASD DC group findings, the overall pattern is surprising given how common language concerns/delays are in emerging ASD. Our lack of findings in the ASD group could reflect: (1) the ASD group is smaller which may leave us with less power to detect an effect if it is there, (2) some of the children within the ASD group did not present with language delays, but, again, our small ASD group did not allow us to consider separately those with and without language delays, and (3) 12 months of age is one of the earliest ages wherein behavioral ASD markers emerge. It could be that dyadic/behavioral differences are not yet apparent in our ASD sample.

With respect to dyadic synchrony and responsiveness, several prospective studies over the past 20 years have also documented distinct group differences (Green et al., 2017; Hudry et al., 2013; Kellerman et al., 2019; Siller & Sigman, 2002; Steiner et al., 2018; Yirmiya et al., 2006). However, once examined by both ASD likelihood and developmental outcome classifications, studies (the present study included) often inconsistently identify systematic differences. This further complicates interpreting the clinical relevance of observed synchrony/responsiveness, making it unclear *how* to best target any observed group differences, while also trying to minimize unnecessary distress/alarm to families. Additional research is needed to elucidate the mixed likelihood and/or outcome group differences and translate them into actionable intervention targets.

### Limitations

Although the exploratory patterns modeled in TVEM highlighted variability in dyadic patterns that would have been missed when only capturing aggregated dyadic differences, this study is not without limitations.

With respect to MR, the majority of previous infant sibling studies report either comparable social exchanges or increases in select prosocial behaviors of parents (e.g., Choi et al., 2020a, 2020b; Choi et al., 2020a, 2020b; Harker et al., 2016; Pijl et al., 2021; Schwichtenberg et al., 2019). However, the present study identified significantly lower odds of MR for the Non-ASD DC group (compared to TYP) for nearly the entire play interaction, as well as during a brief period for the ASD group. Though possible that mothers’ were indeed less responsive to their infants, it is important to note the inherent limitations within our responsiveness composites. Currently to receive credit for an instance of MR, infants must initiate a bid with a target behavior (e.g., look face) to provide an opportunity for mothers to respond. In other words, if infants do not elicit a clear prosocial bid towards their mother, then mothers’ opportunities for exhibiting instances of MR are penalized. Future micro-analytically derived coding schemes should consider better techniques for capturing response-oriented codes that take into account missed vs. unclear vs. lack of opportunities for social response, such as coding the full range of a targeted prosocial behavior (e.g., positive, neutral, or negative affect).

The observed stable patterns of DS, IR, and MR in the present study may also, in part, be due to our coding conventions. First, qualifying instances of DS/IR/MR were simply dichotomized for either having occurred or not as 1,0 (see the full list of qualifying two-event sequences for DS/IR/MR in Table 3). This placed equal weight on the occurrences of DS/IR/MR; however, copious research highlights important distinctions in the *quality* of social bids and that difficulty exhibiting higher-order bids may be more indicative of atypical development (e.g., higher-level vs. lower-level joint attention, Pickard & Ingersoll, 2015); multimodal bids such as social smiling (see reviews, Crowell et al., 2019; Wan et al., 2019). Second, it is also conceivable that we observed relatively stable rates of DS/IR/MR because the coded interaction included only 3 min of play. This may functionally not have been long enough to detect more fluctuations in dyads’ social exchanges. Within this study, the decision to code a 3-min segment was fundamentally a functional one. Future studies could mitigate this limitation with longer segments that naturally start and stop as interactions typically do. Third, though meaningful start times were established in order for all dyads’ instances of DS/IR/MR to occur between 0 and 180 s for the TVEM analyses, start times were based off each dyad’s first prosocial mother or infant behavior. Meaning, it’s quite possible that less engaged dyads first prosocial behavior did not occur within the first several seconds of the coded interaction, which may have limited initial opportunities to capture fluctuations in “slow to warm” dyads. It is also of note that dyadic exchanges can experience change in a wide range of ways; time is only one metric of change. For example, it is possible that rates of change may be meaningfully different across different activities within the 3-min period; this would reflect changes as a function of activity rather than of time. Fourth, mothers were instructed to play with their child for roughly 5 min. Thus, whether the decrease in DS/IR/MR for the Non-ASD DC and TYP groups in the last 20 s of the interaction were due to mothers anticipating the end of the task or a natural lull in the engagement is unknown.

Finally, it is also important to note that TVEM results depicting large confidence interval bands do indicate less precision of the odds ratio (Szumila, 2010). This was especially apparent for the effect of elevated ASD likelihood group for DS (Fig. 4a) and the effect of ASD on DS (Fig. 5a). The wide bands for the elevated ASD likelihood and ASD groups, respectively, are likely indicative of the heterogeneity within their groupings and limited sample sizes. Even though this study included dyadic data from 167 infants across two BSRC sites, sample sizes of 13 for ASD and 33 for Non-ASD DC are too small to further categorize by ASD symptom severity or Non-ASD DC subtype classification. Continued big science efforts are needed to parse these complex intersections.

The covariates explored in this study included only child sex and maternal education, although previous studies note several factors that can influence dyadic synchrony and responsiveness. For example, within this study maternal education attainment was different across EL and TL groups; wherein, mothers in the EL had less education, and this could be a factor in the EL findings. The education variable in this study had several education options that made its incorporation into the models cumbersome and ultimately difficult to interpret. Future studies can build on this covariate weakness by including additional elements like continuous maternal education, maternal mental health (Brookman et al., 2023; Murray et al., 1996), infant sleep (de Graag et al., 2012; Feldman, 2007b), premature birth status (Feldman & Eidelman, 2007; Feldman, 2007a), and stress (Coburn et al., 2015).

### Future Direction

The present study moves the field forward by demonstrating the applicability of TVEMs to social communication skills within the context of elevated ASD likelihood. For similarly coded dyadic interactions, TVEM can easily be applied to existing datasets in order to illustrate the unfolding patterns of select social indices that may provide more context to our current mean-level metrics for characterizing ASD likelihood. In addition, recognizing ongoing efforts to provide infants at elevated ASD likelihood with parent-mediated intervention services within the first year, being able to identify subtle changes in social communication, may better inform how to both capture and measure progress in infants’ development.
